# Response to Food Restriction, but Not Social Information Use, Varies Seasonally in Captive Cardueline Finches

**DOI:** 10.1093/icb/icae016

**Published:** 2024-04-12

**Authors:** J M Cornelius, B J Vernasco, N Mori, H E Watts

**Affiliations:** Department of Integrative Biology, 4575 SW Research Way, Oregon State University, Corvallis, OR 97333, USA; School of Biological Sciences, Washington State University, Pullman, WA 99164 , USA; Department of Biology, Whitman College, Walla Walla, WA 99362, USA; Department of Integrative Biology, 4575 SW Research Way, Oregon State University, Corvallis, OR 97333, USA; School of Biological Sciences, Washington State University, Pullman, WA 99164 , USA; Center for Reproductive Biology, Washington State University, Pullman, WA 99164, USA

## Abstract

Temperate winters can impose severe conditions on songbirds that threaten survival, including shorter days and often lower temperatures and food availability. One well-studied mechanism by which songbirds cope with such conditions is seasonal acclimatization of thermal metabolic traits, with strong evidence for both preparative and responsive changes in thermogenic capacity (i.e., the ability to generate heat) to low winter temperatures. However, a bird’s ability to cope with seasonal extremes or unpredictable events is likely dependent on a combination of behavioral and physiological traits that function to maintain allostatic balance. The ability to cope with reduced food availability may be an important component of organismal response to temperate winters in songbirds. Here, we compare responses to experimentally reduced food availability at different times of year in captive red crossbills (*Loxia curvirostra*) and pine siskins (*Spinus pinus*)—two species that cope with variable food resources and live in cold places—to investigate seasonal changes in the organismal response to food availability. Further, red crossbills are known to use social information to improve responses to reduced food availability, so we also examine whether the use of social information in this context varies seasonally in this species. We find that pine siskins and red crossbills lose less body mass during time-restricted feedings in late winter compared to summer, and that red crossbills further benefit from social information gathered from observing other food-restricted red crossbills in both seasons. Observed changes in body mass were only partially explained by seasonal differences in food intake. Our results demonstrate seasonal acclimation to food stress and social information use across seasons in a controlled captive environment and highlight the importance of considering diverse physiological systems (e.g., thermogenic, metabolic, digestive, etc.) to understand organismal responses to environmental challenges.

## Introduction

Many environments on Earth are characterized by seasonal changes in daylength, temperature, or precipitation (i.e., climate). Seasonal climates in turn drive seasonal patterns in food availability and modulate the costs associated with maintaining allostatic balance (McEwen and Wingfield 2003; Word et al. 2022). Animals living in seasonal environments therefore display phenotypic changes in behavior and physiology that improve survival in the context of seasonal change (Wingfield et al. 1980; Gwinner 1996; Hau 2001; Chmura et al. 2022). While changes in morphology and behavior are often readily observable in animals (e.g., pelage or plumage alteration, hibernation and migration, etc.), physiological changes can be more difficult to detect and often require more detailed experimentation to describe.

Temperate birds are well known, for example, to acclimatize to the cold temperatures associated with winter climates (Hart 1962; Dawson and Marsh 1989; Swanson and Liknes 2006), but they typically must either have predictive cues (e.g., photoperiod) to induce advance preparations or must have time to acclimatize to changing conditions before they can develop such resistance to cold temperatures (Swanson 2010; Swanson and Vézina 2015). It is now understood that many birds undergo changes in enzyme expression and cellular respiration to facilitate a higher thermogenic capacity in anticipation of, or in response to, colder temperatures (Zheng et al. 2008; Liknes and Swanson 2011; Zheng et al. 2014). Such changes allow birds to cope with the colder conditions typical of winter, which are often well below the thermal neutral zone. A growing body of research is dedicated to describing how such changes may influence animal distributions and what the costs and benefits might be for individuals investing in thermogenic cellular machinery (Canterbury 2002; Buckley et al. 2018). For example, heat is often generated by increased shivering or increased activity of futile enzyme cycles (Dawson et al. 1992; Nowack et al. 2017; Li et al. 2019). Such mechanisms may be costly both in terms of energy substrates to power them, and in terms of upsetting oxidative balance or reducing efficiency for other types of cellular work (Kendeigh 1969; Hawley et al. 2012; Stier et al. 2014; Cornelius et al. 2017). Maintenance of body temperature in winter environments, therefore, comes at a higher risk of starvation, especially given that food resources are often less predictable or of lower quality (Ketterson and Nolan Jr. 1982; Rogers 1987; Brodin 2007). We therefore hypothesize that temperate birds may possess other forms of physiological acclimatization that improve survival when conditions are challenging and food resources are less predictable.

Specifically, birds living in seasonal environments may be more robust to food resource limitation in winter and exhibit behavioral or physiological responses that improve resilience when food intake is limited. This is suggested by two previous studies: rufous hummingbirds (*Selasforus rufus*) utilize torpor to preserve body mass during extreme food restriction in spring, but not summer (Hiebert 1991), and European starlings (*Sturnus vulgaris*) limited to 6 h to feed each day carry more mass when housed on a late winter photoperiod but not when housed on a summer photoperiod (Witter and Cuthill 1993). These studies suggest that strategies for coping with energy challenges differ across seasons and that birds may be more responsive to starvation risk in winter. Many birds carry more fat in winter (King and Farner 1966; Blem 1976) and birds living in places with very cold winters may have larger intestines compared to summer (Pendergast and Boag 1973; Liknes and Swanson 2011; Lv et al. 2014; Petit et al. 2014; Wu et al. 2014), though this pattern is not apparent in all temperate species as house sparrows (*Passer domesticus*) in one study did not (Liknes and Swanson 2011). While intestinal tissue is considered expensive to maintain and may impact basal metabolic rate in vertebrates (Cant et al. 1996; Mueller and Diamond 2001), larger intestines also improve absorption of nutrients (Karasov and Diamond 1983; Obst and Diamond 1992; Cant et al. 1996)—suggesting that intestinal mass may help fuel the high-energy turnover typical of winter and buffer against temporary declines in food. In fact, some vertebrates maintain or increase intestinal mass when food is lightly or moderately restricted (Swatson et al. 2002; Chappell et al. 2003). More severe restrictions, however, can lead to reduction of intestinal tissue—presumably due to trade-offs between maintenance of gut versus other tissues that may arise during severe energy deficit (Lee et al. 2002; Chappell et al. 2003; Karasov et al. 2004). Intestinal mass has also been found to be sensitive to social information in the context of food restriction. Social information refers to information gathered through observation of other individuals behaviors (e.g., acoustic or visual cues) or the products of their behavior (Valone and Templeton 2002). In red crossbills (*Loxia* curvirostra), social information from food-restricted conspecifics has been found to improve outcomes in individuals experiencing food restriction, including the conservation of body mass and intestinal mass following a severe restriction (Cornelius 2022). Social information appears to impart these effects through integrated, coordinated responses in the brain and gut—perhaps via the hypothalamic-pituitary-adrenal (HPA) axis, which is also responsive to social information about food availability (Cornelius et al. 2010; Cornelius et al. 2018). Together, these findings suggest that social information may help birds respond to food limitations by improving assimilation capacity and sensitizing the endocrine response to stress. Such responses to social information could be especially adaptive in winter, when food is more limited and thermoregulatory costs are higher, but studies specifically examining seasonal responses to food restriction are few (Hiebert 1991; Witter et al. 1995).

Here, we experimentally test the hypothesis that songbirds cope better with food restriction in winter than in summer using two species of finch that specialize on unpredictable food sources and winter at high latitudes and altitudes in the north temperate zone. This hypothesis predicts that mass loss will be lower in winter compared to summer when experiencing the same food restriction. We also evaluate whether responsiveness to predictive social information varies across seasons to provide one of the first assessments of seasonal changes in social information use. Birds may be similarly responsive to predictive social information across seasons, or, alternatively, responsiveness to predictive social information may be enhanced in the winter, indicated by improved outcomes (e.g., lower mass loss during restriction) with predictive information in the winter compared to the summer.

## Methods

We performed paired experiments in summer and late winter to investigate the impact of food restriction on body mass in two species of finch, the red crossbill and the pine siskin (*Spinus pinus*). All four experiments were identical with respect to their food-restriction treatment: birds were limited to two, 45-min feeding sessions per 24-h period and were measured for changes in body mass across 1–3 days of restriction (Wurtz et al. 2021; Cornelius 2022). In red crossbills, this food-restriction experiment was conducted both with and without predictive social information about declining food. Predictive social information about declining food availability was provided by housing focal birds with neighbors that experienced food restriction for 3 days prior to a focal bird experiencing the food restriction (Cornelius 2022). Birds without predictive social information had well-fed neighbors before the restriction period. Both groups (i.e., focal birds with and without social information) had well-fed neighbors during the restriction period. In combination, these experiments allow us to assess if coping ability during food restriction and the use of social information changes across seasons. All methods were approved by the Oregon State University Institutional Animal Care and Use Committee.

### Focal species

The red crossbill is a nomadic species of Cardueline finch that specializes on conifer seeds and can flexibly breed in winter and summer when food is abundant (Hahn et al. 2008). There are different morphs, called eco-types or types, of red crossbill that vary in bill depth, body size, and call structure, and individuals are assigned to a particular “eco-type” or “type” based on their calls (Groth 1993; Benkman and Young 2020). In these experiments, we used type 2 red crossbills, which are one of the larger crossbill eco-types that frequently feed on a variety of pines, spruces, and Douglas fir conifers (Benkman and Young 2019). Type 2 crossbills were captured at various field sites in the east Cascade mountain range of Oregon and Washington, USA. Closely related to the red crossbill, the pine siskin is also a nomadic Cardueline finch. Pine siskins also frequently feed on conifer seed crops, but they are more generalist in diet than are the crossbills, particularly when other seeds are abundant in spring and summer (Dawson 2020). Pine siskins in these studies were captured at various field sites in the east Cascade, Blue, or coastal mountain ranges of Oregon and Washington, USA. All individual crossbills and siskins had acclimated to captivity for at least 9 months prior to each experiment. Experimental histories are given below for birds in each experiment separately, but all birds were naïve to food restriction since entering captivity and prior to the start of the experiments.

### Experiment timing, housing, and captive bird history

All individuals were held on a photoperiod mimicking natural daylength for Corvallis, OR, USA, using full-spectrum lights and temperature was maintained between 18 and 22°C in all experiments. Summer experiments occurred in mid-late July (15.5 L:8.5 D) and winter experiments occurred in early-mid March (11.5 L:13.5 D). We categorize March here as “late winter” because the experiment occurred before the equinox and because conditions in higher elevation coniferous forests of North America, where these species typically occur, are often still well below the thermal neutral zone and prone to snow. Birds were maintained in groups in aviaries (0.75 × 2 × 2 m) prior to the experiments and were acclimated to experimental cages (0.4 × 0.3 × 0.2 m) for at least 3 weeks prior to the start of food restriction to allow body mass to stabilize. Crossbills were maintained on Roudybush pellet food (Roudybush maintenance diet; Woodland, CA, USA) and were given a daily seed allotment of three sunflower seeds. Pine siskins had access to Roudybush at all feeding times and received one teaspoon of a nyjer and sunflower seed mixture daily. Diets were identical across seasons. Birds were housed in a paired-cage configuration that allowed visual access to one immediate neighboring cage only, though they could hear the other individuals in the room, which included other birds in their own treatment group and that of their neighbors (described below for each experiment). Birds in these pairings were of the same sex as determined by phenotype [crossbill feather plumage (Pyle 2022) and siskin reproductive morphology] or genotype (siskins only) to reduce potential differences in reproductive condition. Pine siskins were genetically sexed using DNA extracted from blood samples by the Washington Animal Disease Diagnostic Laboratory (Çakmak et al. 2017).

Experiment 1 (July, 2020): Red Crossbill (*N* = 24 adult females, 18 adult males). The individuals in this experiment had previously been used as controls in another study in which they were held under standard housing conditions and measures of migratory behavior and physiology were periodically collected. The food restriction study began in July. The birds were kept in the same housing configuration as in the prior experiment to avoid disrupting body mass or behavior, which can occur when birds are transferred to different cages (JMC unpublished data). The birds were randomly assigned to a paired-cage configuration as described above, where one cage housed two crossbills and the other housed a single crossbill, all of the same sex. We assigned the cage that housed two birds to the treatment group without predictive social information (i.e., restricted first) and the cage housing one bird to the treatment group with predictive social information (i.e., restricted second). Social information about declining food therefore came from the neighboring birds because they were food restricted first (see below for details about food restriction). The data from experiment 1 are a subset of data that were previously published (Cornelius 2022).

Experiment 2 (early-mid March, 2021): Red Crossbill (*N* = 12 adult females, 12 adult males). These individuals had not previously been used in any experiment. Crossbills were randomly assigned to same-sex pairs and housed singly next to each other in the same paired-cage configuration as described above. One bird in each pair was assigned to the treatment group without predictive social information and the other to the treatment group with predictive social information. Crossbills in this experiment could hear other siskins and crossbills in the room that were experiencing an identical restriction protocol.

Experiment 3 (early-mid March, 2021): Pine Siskin (*N* = 6 adult females, 6 adult males). These individuals had not previously been used in any experiment. All siskins in this experiment were restricted on the same time schedule and did not receive predictive social information from any other birds. They were housed singly next to a well-fed crossbill (i.e., with food *ad libitum*) prior to and during food restriction. They could hear other food-restricted siskins and well-fed crossbills in the room.

Experiment 4 (July, 2023): Pine Siskin (*N* = 5 adult females, 7 adult males). These individuals had not previously been used in any experiment. All siskins that experienced food restriction in this experiment were restricted on the same time schedule and did not receive predictive social information from any other birds. Birds in this experiment were housed singly next to a well-fed pine siskin neighbor. Birds in this experiment could hear other food-restricted and well-fed siskins in the room. They could also hear (but not see) other birds housed in the room, including well-fed crossbills and a mix of restricted and well-fed house finches that were on the same restriction schedule as the siskins. House finches were not included in this study because they have not yet been experimentally restricted in winter.

## Food restriction protocol

Each of the four experiments described above used a common time-restricted feeding paradigm to generate a food restriction (Cornelius 2022). Food restriction began on Day 0, ∼4 h after lights-on, when all food cups were removed from the cage and birds were measured for mass. Birds then received two feeding sessions per 24-h period, one in the afternoon and the other in the morning. Each feeding session lasted 45 min, during which they had access to their food cups containing at least 10 times the normal daily intake of food (i.e., food was provided *ad libitum* during the feeding sessions). Feeding sessions occurred at variable times during the first 2 h of lights-on for morning sessions and during the last 4 h of lights-on for afternoon sessions. Food restriction continued for up to 3 days or until birds lost 10% body mass, after which they were returned to unlimited food access. On days 1 and 3, body mass was measured. Morning feeding sessions were scheduled such that there was at least 2 h after the feeding session ended before mass measurements were collected, thus allowing for full evacuation of the gut and stabilization of body mass prior to measurements (Cornelius 2022). Timing of feeding and body mass measurements were identical for crossbills with and without predictive social information. Food intake was measured for each 24-h period of restriction as the difference in mass between food provided and food remaining in a covered cup using an Ohaus electronic balance to the nearest hundredth of a gram.

### Data analysis

Data from red crossbills and pine siskins were analyzed separately. For pine siskins, the experimental groups included summer food restriction and winter food restriction. All pine siskins experienced the food restriction without predictive social information. For red crossbills, experimental groups included summer food restriction with and without predictive social information and winter food restriction with and without predictive social information. Thus, season and social information were administered in a full factorial design in red crossbills.

Sex did not influence mass loss during food restriction in past experiments (Cornelius et al. 2010; Cornelius et al. 2018), nor were there differences in this study (Cornelius 2022; Student’s *t*-test *P* > 0.3 for all treatment groups); thus, sex was not considered in analyses. Initial body mass and food intake were compared across seasons for each treatment group using an ANOVA (red crossbills with and without predictive social information) or Student’s *t*-test (pine siskins) to determine if birds started in different conditions in summer versus winter.

Percent change in body mass and % change in food intake were calculated for each individual following 1 and 3 days of time-restricted feeding. In siskins, the % changes in body mass and food intake during food restriction were compared between seasons using a Student’s *t*-test. Residuals for body mass and food intake were normally distributed and passed Levene’s test for homogeneity of variance (*P* = 0.17–0.12, respectively).

To determine if the response to food restriction or the protective effect of predictive social information changed seasonally in crossbills, we compared the % loss in body mass and food intake at days 1–3 using linear mixed models fit by restricted maximum likelihood with season (categorical), predictive social information (categorical), and their interaction as fixed effects. Individual identity was included as a random effect. Raw data and residuals were normally distributed with skewness <1 and kurtosis <2 for all distributions (Kim 2013).

To determine whether changes in food intake predicted the change in body mass, we compared the two metrics using linear regression at days 1 and 3 for each species separately.

## Results

Initial body mass and food intake did not vary across seasons in captive red crossbills with or without predictive social information (ANOVA *P* = 0.3; all seasonal pairwise comparisons *P* > 0.2), but pine siskins in summer started with higher body mass and food intake compared to winter (body mass *P* = 0.04, winter average 13.0 g +/−0.8 SEM, summer average 15.2 g +/−0.9 SEM; food intake *P* = 0.003, winter average 2.8 +/−0.4 SEM, summer average 4.4 +/−0.4 SEM).

### Siskin seasonal comparison

**Fig. 1:**
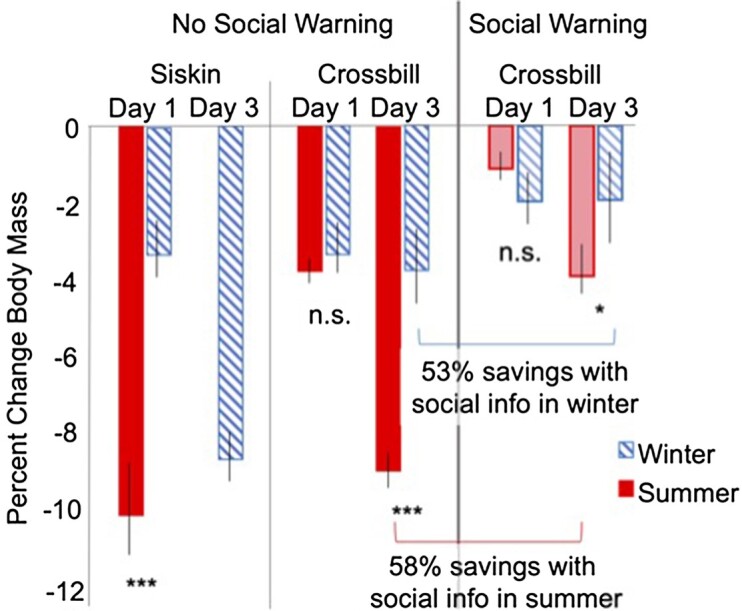
Red crossbills and pine siskins lost more body mass in summer (solid bars) compared to winter (hatched bars) when restricted in the total amount of time available to feed. Red crossbills that were given predictive social information about declining food (lighter shades; right panel) prior to the food restriction saved approximately 53% of the mass lost after three days of restriction in winter compared to 58% in summer (comparison shown with brackets), which was a weak trend in the linear mixed model (*P* = 0.08). Asterisks denote mass losses in summer that are larger than the equivalent restriction day for that species in winter by Student’s t-test (siskins) or linear mixed model estimates (crossbill)(*P < 0.05; ***P < 0.0001). Pine siskins in summer lost mass so rapidly that the food restriction was terminated early, thus there are no Day 3 data provided for siskins in summer.

**Fig. 2:**
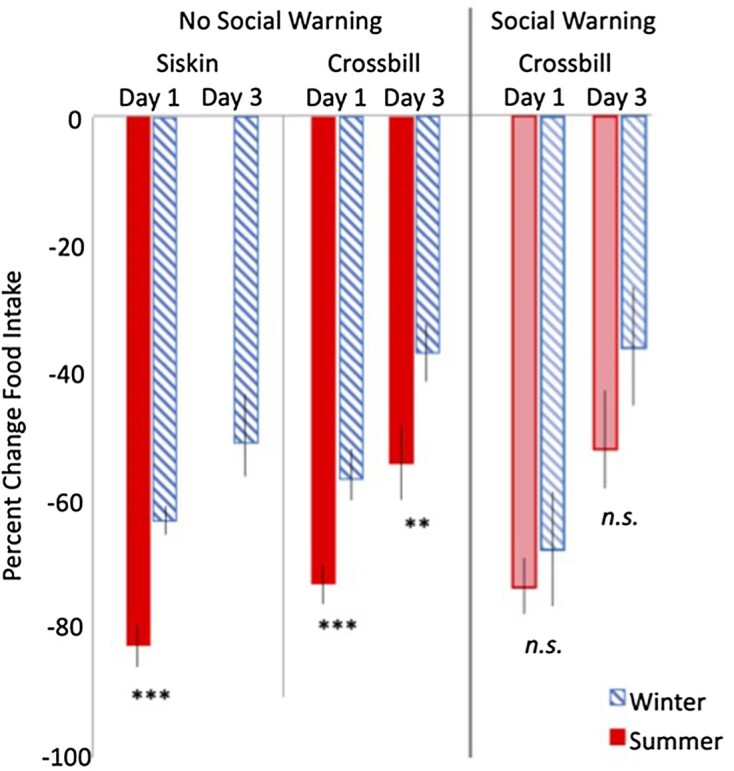
Crossbills and siskins without predictive social information experienced a larger percent reduction in food intake during time-restricted feedings in summer (solid bars) compared to winter (hatched bars). Crossbills with predictive social information, however (right panel), showed no significant differences in food intake between summer and winter; this may be due in part to higher variability in intake. Asterisks denote mass losses in summer that are larger than the equivalent restriction day for that species in winter by Student’s t-test (siskins) or linear mixed model estimates (crossbill)(**P < 0.005; ***P < 0.0001). There are no food intake data for pine siskins on day 3 in summer because their restriction was terminated early.

Pine siskins had a larger % reduction in food intake and body mass in summer compared to winter after 1 day of time-restricted feeding (Student’s *t* = 5.96; *P* < 0.0001 and *t* = 5.4; *P* < 0.0001, respectively; Figs 1 and 2). These also reflected larger absolute reductions; the average change in food intake after 1 day of restriction was −3.9 g +/−0.2 SEM in summer and −1.8 g +/−0.1 SEM in winter, and the average change in body mass was −1.5 g +/−0.2 in summer and −0.45 g +/−0.07 in winter. Restriction in summer was halted after 1 day due to concerns over the death of one individual and because more than half of the remaining individuals had lost >10% body mass. We therefore do not compare Day 3 mass losses in siskins but still show Day 3 data for winter siskins. No winter siskins lost >10% body mass in the experiment at Days 1 or 3.

### Crossbill seasonal comparison

No red crossbills lost >10% body mass in the experiment. Red crossbills lost similar amounts of body mass after 1 day of time-restricted feeding across the two seasons (Season [Summer] $\beta \ $= 0.05, *P* = 0.78; Table 1; Fig. 1), despite having a larger % reduction in food intake after 1 day of restriction in summer compared to winter (Season [Summer] $\beta $ = −6.1, *P* < 0.0001; Table 2; Fig. 2). The average change in food intake after 1 day of restriction was −5.4 g +/−0.3 SEM in summer and −3.4 g +/−0.3 SEM in winter, and the average change in body mass was −1.4 g +/−0.1 in summer and −1.2 g +/−0.2 in winter. The effect of predictive social information was also similar across seasons after 1 day of restriction for body mass (Social Information [None] *Season [Summer] $\beta $ = −0.22; *P* = 0.17; Table 1), though there was a weak trend for food intake to be higher in summer (Social Information [None] *Season [Summer] $\beta $ = −3.6; *P* = 0.09; Table 2). The average change in food intake after 1 day of restriction in red crossbills with predictive social information was −5.2 g +/−0.6 SEM in summer and −4.4 g +/−0.6 SEM in winter, and the average change in body mass was −0.5 g +/−0.2 in summer and −0.7 g +/−0.2 in winter.

**Table 1:** LMM output explaining the % change in body mass after 1 and 3 days of time-restricted feedings in red crossbills with and without predictive social information.

*Day 1 – Body mass*			
*Parameter*	*Estimate*	*t ratio*	*P*
Season [Summer]	0.05	0.27	0.79
**Predictive social information [None]**	−**0.99**	−**4.94**	**<0.0001**
Predictive social information [None] *Season [Summer]	−0.27	−1.37	0.17
***Day 3 – Body mass***			
*Parameter*	*Estimate*	*t ratio*	*P*
**Season [Summer]**	−**1.95**	−**5.01**	**<0.0001**
**Predictive social information [None]**	−**1.86**	−**4.80**	**<0.0001**
Predictive social information [None] *Season [Summer]	−0.69	−1.78	0.08

**Table 2:** LMM output explaining the % change in food intake after 1 and 3 days of time-restricted feedings in red crossbills with and without predictive social information.

*Day 1 – Food intake*			
*Parameter*	*Estimate*	*t ratio*	*P*
**Season [Summer]**	−**6.09**	−**2.91**	**<0.0001**
Predictive social information [None]	3.06	1.46	0.15
Predictive social information [None] *Season [Summer]	−3.61	−1.72	0.09
***Day 3 – Food intake***			
*Parameter*	*Estimate*	*t ratio*	*P*
**Season [Summer]**	−**9.38**	−**3.13**	**0.003**
Predictive social information [None]	−1.68	−0.56	0.58
Predictive social information [None] *Season [Summer]	−1.43	−0.48	0.63

However, after 3 days of restriction, there emerged strong seasonal differences in both food intake and mass loss (Figs 1 and 2). Crossbills that were restricted in summer ate less (Season [Summer] $\beta $ = −11.9, *P* = 0.003) and lost more body mass compared to those restricted in winter at day 3 (Season [Summer] $\beta $ = −1.95; *P* < 0.0001). The average change in food intake after 3 days of restriction was −4.3 g +/−0.3 SEM in summer and −2.3 g +/−0.3 SEM in winter, and the average change in body mass was −3.3 g +/−0.2 in summer and −1.4 g +/−0.3 in winter. There was a weak trend for predictive social information to have a slightly larger protective effect on body mass in summer after 3 days of time-restricted feedings compared to winter (Social Information [None] *Season [Summer] $\beta $ = −0.69, *P* = 0.08), but the protective effect was present in both seasons and the seasonal difference in mass savings was small (i.e., 58% mass savings in summer versus 53% mass savings in winter with predictive social information). Social information did not influence the % change in food intake within or across seasons throughout the period of food restriction (Table 2). The average change in food intake for red crossbills with predictive social information after 3 days of restriction was −3.9 g +/−0.7 SEM in summer and −2.6 g +/−0.7 SEM in winter, and the average change in body mass was −1.5 g +/−0.3 in summer and −0.6 g +/−0.3 in winter.

### Food intake versus mass change

**Fig. 3:**
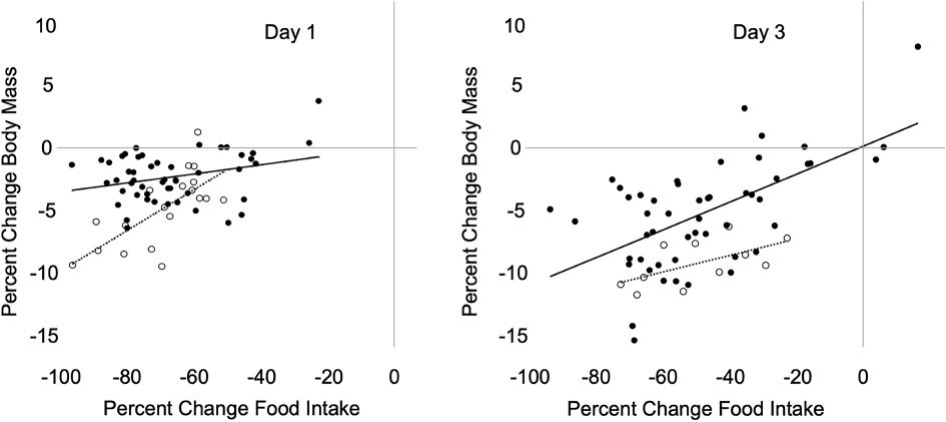
The % change in food intake does not fully explain changes in body mass in red crossbills and pine siskins when given limited time to feed. Lines show best fit by linear regression in red crossbills (solid, closed circle) and pine siskins (dashed, open circle). After 1 day of food restriction (left panel), the change in food intake explained only 8% of the change in body mass of crossbills (*P* = 0.03, *R*^2^ = 0.08) and 49% in siskins (*P* = 0.0008, *R*^2^ = 0.49). After 3 days of food restriction (right panel), the change in food intake explained ∼35% of the change in body mass for both species (crossbill *P* < 0.0001, *R*^2^ = 0.35; siskin *P* = 0.05, *R*^2^ = 0.35), but note that data on Day 3 for siskins included only those sampled in winter.

The % change in food intake positively predicted the % change in body mass after 1 or 3 days of restriction in both red crossbills and pine siskins—where birds with a larger % reduction in food intake had a larger loss of body mass (linear regressions: Day 1 crossbill *P* = 0.03, siskin *P* = 0.0008; Day 3 crossbill *P* < 0.0001, siskin *P* = 0.05; Fig. 3). However, changes in food intake explained a relatively small amount of the variance in body mass change in crossbills (Day 1 *R*^2^ = 0.08 and Day 3 *R*^2^ = 0.35) and less than half of the variance in siskins (Day 1 *R*^2^ = 0.49 and Day 3 *R*^2^ = 0.35).

## Discussion

Captive red crossbills and pine siskins were better able to conserve body mass during time-restricted feedings imposed in late winter compared to summer. While differences in food intake partially explained the mass savings observed in winter, there are likely other processes at work that improve the ability of wintering finches to cope with reduced food availability. These results support the hypothesis that birds may be more robust to food limitation in winter and exhibit behavioral or physiological responses that improve resilience when food is limited. The mechanisms underlying this resilience may be regulated by photoperiod or circannual rhythms given that temperature and food cues were identical between seasons. Although not examined in this study, low temperatures could provide an additional cue that might enhance resilience to food limitation and testing these species earlier in the winter when average temperatures are colder may reveal finer-scale seasonal patterns. We also found that social cues mitigate body mass loss during restriction in winter, in agreement with previous work conducted in summer (Cornelius 2022), though it is important to determine if such information is utilized in free-living systems given the captive nature of these experiments. Further, distinguishing what social cues are utilized by birds and whether or not the response is specific to the context of food stress would help to determine if the social information involves specific or intentional signaling. Regardless, the protective effect of social information did not differ between seasons, highlighting that social information is relevant throughout the year. This suggests that the mechanisms that allow for better conservation of body mass in winter and in response to social information are either distinct from each other or can be amplified when they occur in coincidence.

The time-restricted feeding in this experiment caused an average reduction in food intake of ∼85 and 75% in summer for pine siskins and red crossbills, respectively, compared to ∼60% for both species in winter, when social information from restricted conspecifics was absent. Severe food restrictions that cause decreases in food intake of >50% are known to cause reductions in the size of the intestine and other organs in birds, and these reductions in tissues can delay the ability of birds to gain mass even when food availability is no longer limited (Klaassen and Biebach 1994; Karasov and Pinshow 2000; Karasov et al. 2004; Pierce and McWilliams 2004; Zhang et al. 2018). If the difference in food intake observed in this study differentially impacted intestinal mass, this may in part explain the imperfect relationship between % change in food intake and body mass loss—as larger intestines would improve absorption and assimilation of nutrients from the food (Karasov and Diamond 1983; Obst and Diamond 1992).

Intestines can respond to cues other than food volume, such as quality of food and social cues. For example, European starlings fed identical amounts of lower-quality food developed larger intestines relative to those with high-quality food (Geluso and Hayes 1999), and red crossbills with predictive social information from food-restricted neighbors had larger intestinal mass after food restriction compared to those without (Cornelius 2022). In the red crossbill study, impacts of social information may have occurred either via protection of intestinal mass from atrophy during the actual food restriction, or by inducing the intestine to grow prior to the restriction period when social cues were present. Regardless of the mechanisms, a larger intestine could facilitate coping with reduced food availability and may help explain the greater mass savings observed in the predictive social information group compared to crossbills without warning on day 1, despite relatively similar changes in food intake between those groups. We did not measure intestinal mass in the winter experiments, and so we were unable to say if the seasonal mass savings observed in this experiment share a similar mechanism of flexible intestinal morphology, but some free-living birds inhabiting highly seasonal environments show winter increases in the size of the intestine and the expression of enzymes that regulate absorption (Zheng et al. 2008; Olsen et al. 2011; Lv et al. 2014). We have also observed that captive red crossbills held in constant cold temperatures have larger intestines in late winter compared to those held at warmer temperatures during the same time of year (JMC unpublished data), as is the case in some other species (Williams and Tieleman 2000). Together, these studies suggest that birds may flexibly adjust intestinal morphology in response to food, social cues, and climate. Such mechanisms may be critically important in balancing the high energy demands of winter with the less predictable or lower quality food that is common for many species that winter in seasonally cold climates.

Pine siskins and red crossbills showed similar patterns in seasonal responses to food restriction, but there were some interesting differences. Red crossbill body mass was more resilient to change in food supply than was pine siskin body mass during the early phases of food restriction in summer despite a large decrease in food intake. Pine siskins, on the other hand, started with higher body mass in summer compared to winter, yet they were not as tolerant of the food restriction in summer. In fact, we terminated the restriction after 1 day because one bird died and others dropped body mass so quickly that we were concerned about their ability to survive for another restriction day. These data suggest that while both species are less resilient to food restriction in summer compared to winter, pine siskins may be even more at risk to unpredictable changes in food availability—perhaps because they are less than half the size of the red crossbills used in this study. Smaller-bodied animals have higher mass-specific metabolic rates (Kleiber 1947; Nagy 2005); thus, a % change of food intake for a smaller animal may have a comparatively larger impact on mass (Ketterson and King 1977; Nagy 1987). Smaller-bodied birds may therefore face particularly strong selection for seasonal coping mechanisms against starvation.

Small birds appear to employ a variety of approaches to defend against energy loss when food intake declines. These approaches include heterothermy (Hohtola 2012), ptiloerection (Morris 1956), and carrying extra energy reserves (i.e., body mass or fat). For example, rufous hummingbirds can apparently compensate for substantial reductions in food intake with torpor in winter, and this is so effective at reducing energy costs that body mass can actually increase even when restricted by 90% normal intake (Hiebert 1991). These hummingbirds do not, however, utilize similar strategies when they are food restricted in summer and body mass therefore declines—suggesting that compensatory mechanisms cannot be or are not utilized similarly at all times of year (Hiebert 1991). White-crowned sparrows (*Zonotrichia leucophrys*) also use heterothermy when exposed to cold temperatures and do so to a greater degree when fasted (Ketterson and King 1977). European starlings (*Sturnus vulgaris*), given food for only part of the day (i.e., a 6-h deprivation), responded by carrying extra body mass when maintained on a late winter photoperiod but did not carry extra body mass on a late summer photoperiod (Witter et al. 1995). These findings suggest that birds are programmed to respond to changes in food differently across seasons—perhaps because of the higher starvation risk that the winter season typically carries or requirements of different life history stages (e.g., breeding or molt). For red crossbills and pine siskins, we don’t know if heterothermy is used to cope with food restrictions in either season. But the near-relative American goldfinch, which is similar in size to the siskin, is known to utilize a reduced body temperature to survive cold winter nights (Lustick et al. 1982), whereas crossbills may not (Dawson and Harrison 1964).

Carrying additional energetic reserves in the winter may also be a strategy employed by free-living pine siskins and American goldfinches, which show seasonal peaks in subcutaneous body fat stores in the winter (Cornelius et al. 2021). Red crossbills, however, often breed in winter, which may limit their ability to carry large fat stores, and their average fat deposits in free-living populations do not peak until the late spring migratory period in May and June (Cornelius and Hahn 2012; Cornelius et al. 2021). Thus, pine siskins and red crossbills may be impacted by seasonal patterns of food availability differently and may use somewhat different approaches to cope with reduced food, which could underlie the differences observed between siskins and in response to the experimental food restriction. It is also possible that differences in breeding condition could generate differences in response to food restrictions despite individual housing and same-sex pairing. The late winter and summer experiments occurred during the middle and start of the winter and summer red crossbill breeding seasons, respectively, and during the start and end of the siskin breeding season, respectively (Benkman and Young 2020; Dawson 2020); however, we note that none of the females in these experiments exhibited brood patches indicative of a breeding state. Finally, another possibility is that red crossbills are better adapted to coping with unpredictable food in summer compared to pine siskins because their higher degree of specialization on conifer seed crops makes them less flexible when conifer seed crops fail compared to pine siskins—which have a much larger array of possible food sources available to them in summer (Benkman and Young 2020; Dawson 2020).

There were slight variations in housing conditions across the experiments that should be considered in interpreting the results. Pine siskins in winter could see well-fed red crossbills whereas pine siskins in summer could see well-fed pine siskins, though birds in both experiments could hear both pine siskins and red crossbills. Further, pine siskins in summer could hear both well-fed and food-restricted pine siskins and house finches, whereas pine siskins in winter could only hear food-restricted pine siskins and well-fed red crossbills. But evidence from three other experiments suggests these differences in social housing are unlikely to underlie the observed changes in body mass. One prior experiment found that, relative to conspecific presence, heterospecific presence (i.e., the red crossbill) had no stimulatory effect on body mass or reproductive development during the migratory-breeding transition (i.e., April−June) in pine siskins (Vernasco et al. 2024). Further, social information about declining food from heterospecifics in another experiment did not affect mass loss during food restriction in red crossbills (JMC unpublished data). Third, pine siskins that could hear but not see a mix of well-fed and food-restricted conspecifics housed in the same room had similar changes in body mass during a moderate food restriction as those who could hear only food-restricted conspecifics (Robart and Watts 2023). Thus, the presence of heterospecifics or some well-fed pine siskins in the room during the summer experiment is unlikely to have generated the seasonal body mass responses to restriction that were observed in siskins in this study. Red crossbills also had a slight variation in social environment in the summer compared to the winter experiment, because there were two birds per cage in the group without predictive social information in summer. Double-housed birds may have experienced different feeding dynamics during time-restricted trials in summer. However, we believe this is likely not the case for several reasons. First, we supplied multiple food cups in the cages housing two birds and did not observe exclusion behaviors. Second, the birds completed their feeding behavior prior to the termination of each timed trial (JMC personal observation)—suggesting that they stopped eating due to internal mechanisms rather than simply running out of time to eat during the 45-min sessions. Third, patterns of mass loss across seasons were similar for both double- and single-housed birds. Thus, our overall interpretation is that the seasonal differences that were observed in response to time-restricted feeding in this experiment are not likely explained by these differences in housing conditions.

We have found that response to severe time-restricted feeding is sensitive to both season and social information in Cardueline finches. These differences in response may be supported by flexible changes in gut morphology, feeding behavior, or other unidentified mechanisms (e.g., hypothalamic gene expression; Mercer et al. 2001). The hypothalamic-pituitary-adrenal axis is positioned to be an important mediator of flexible changes in behavior and physiology in response to seasonal and social cues. Prior research has found that social information from food-restricted neighbors has strong impacts on receptor expression in brain regions that modulate the stress response (Cornelius et al. 2018), and the response to stress is known to be seasonally modulated in many vertebrate species (Romero 2002). Thus, research investigating links between the hypothalamic-pituitary-adrenal axis and the gut across seasons, social contexts, and fluctuations in weather could be especially illuminating. It is important to understand these mechanisms, as they may be important for survival when food becomes limited and are probably costly to maintain given that they are apparently down-regulated in summer when food is typically more abundant. The results presented here highlight the potential importance of considering behavioral and physiological traits, beyond those directly related to thermogenic capacity, in advancing our understanding of how animals cope with seasonal environments.

## Data Availability

Data are available in Dryad: https://doi.org/10.5061/dryad.37pvmcvt6.

## Notes

From the symposium “Recent advances in the mechanistic understanding of avian responses to environmental challenges” presented at the virtual annual meeting of the Society for Integrative and Comparative Biology, January 2–6, 2024.

Cover Photo Submission Credit: Jamie Cornelius

## References

[bib1] Benkman C, Young M. 2019. Red crossbill (*Loxia curvirostra*), version 2.0. In: Rodewald P. G., editor. The birds of North America. Ithaca (NY): Cornell Lab of Ornithology.

[bib2] Benkman CW, Young MA. 2020. Red crossbill (*Loxia curvirostra*). In: Billerman B. K. K. S. M., Rodewald P. G., Schulenberg T. S., editors. Ithaca (NY): Birds of the World Cornell Lab of Ornithology.

[bib3] Blem CR . 1976. Patterns of lipid storage and utilization in birds. Am Zool. 16:671–84.

[bib4] Brodin A . 2007. Theoretical models of adaptive energy management in small wintering birds. Phil Trans R Soc B. 362:1857–71. 17827099 10.1098/rstb.2006.1812 PMC2442386

[bib5] Buckley LB, Khaliq I, Swanson DL, Hof C. 2018. Does metabolism constrain bird and mammal ranges and predict shifts in response to climate change?. Ecol Evol. 8:12375–85. 30619552 10.1002/ece3.4537 PMC6308872

[bib6] Çakmak E, Akın Pekşen Ç, Bilgin CC. 2017. Comparison of three different primer sets for sexing birds. J VET Diagn Invest. 29:59–63. 28074715 10.1177/1040638716675197

[bib7] Cant JP, Mcbride BW, Croom WJ. 1996. The regulation of intestinal metabolism and its impact on whole animal energetics. J Anim Sci. 74:2541–53. 8904723 10.2527/1996.74102541x

[bib8] Canterbury G . 2002. Metabolic adaptation and climatic constraints on winter bird distribution. Ecology. 83:946–57.

[bib9] Chappell VL, Thompson MD, Jeschke MG, Chung DH, Thompson JC, Wolf SE. 2003. Effects of incremental starvation on gut mucosa. Dig Dis Sci. 48:765–9. 12741469 10.1023/a:1022849112100

[bib10] Chmura HE, Schultz EM, Brazeal KR, Watts HE, MacDougall-Shackleton SA, Hahn TP, Cornelius JM. 2022. Annual schedules. In: Sturkie’s avian physiology. Amsterdam: Elsevier. p. 1183–210.

[bib11] Cornelius EA, Vézina F, Regimbald L, Hallot F, Petit M, Love OP, Karasov WH. 2017. Chickadees faced with unpredictable food increase fat reserves but certain components of their immune function decline. Physiol Biochem Zool. 90:190–200. 28277950 10.1086/689913

[bib13] Cornelius JM, Breuner CW, Hahn TP. 2010. Under a neighbor’s influence: public information about food affects hormones and behavior of a songbird. Proc R Soc Biol Sci. 277:2399–404. 10.1098/rspb.2010.0164 PMC2894903 20356895

[bib15] Cornelius JM, Hahn TP, Robart AR, Vernasco BJ, Zahor DL, Glynn KJ, Navis CJ, Watts HE. 2021. Seasonal patterns of fat deposits in relation to migratory strategy in facultative migrants. Front Ecol Evol. 9:419.

[bib14] Cornelius JM, Hahn TP. 2012. Seasonal pre-migratory fattening and increased activity in a nomadic and irruptive migrant, the red crossbill *Loxia curvirostra*. Ibis. 154:693–702.

[bib16] Cornelius JM, Perreau G, Bishop VR, Krause JS, Smith R, Hahn TP, Meddle SL. 2018. Social information changes stress hormone receptor expression in the songbird brain. Horm Behav. 97:31–8. 29030109 10.1016/j.yhbeh.2017.10.002 PMC5780353

[bib12] Cornelius JM . 2022. Advance social information allows red crossbills (*Loxia curvirostra*) to better conserve body mass and intestinal mass during food stress. Proc Biol Sci. 289:20220516. 35582792 10.1098/rspb.2022.0516 PMC9114945

[bib17] Dawson A . 2020. Pine Siskin (*Spinus pinu*s). In: Poole A. F., editor. Birds of the world. Ithaca (NY): Cornell Lab of Ornithology.

[bib18] Dawson WR, Carey C, Van't Hof TJ. 1992. Metabolic aspects of shivering thermogenesis in passerines during winter. Ornis Scandinavica. 23:381–7.

[bib19] Dawson WR, Marsh RL. 1989. Metabolic acclimatization to cold and season in birds. In: Physiology of cold adaptation in birds. New York (NY): Springer. p. 83–94.

[bib20] Dawson WR, Tordoff HB. 1964. Relation of oxygen consumption to temperature in the red and white-winged crossbills. The Auk. 81:26–35.

[bib21] Geluso K, Hayes JP. 1999. Effects of dietary quality on basal metabolic rate and internal morphology of European starlings (*Sturnus vulgaris*). Physiol Biochem Zool. 72:189–97. 10068622 10.1086/316654

[bib22] Groth JG . 1993. Evolutionary differentiation in morphology, vocalizations, and allozymes among nomadic sibling species in the North American red crossbill. Berkeley (CA): University of California Press.

[bib23] Gwinner E . 1996. Circadian and circannual programmes in avian migration. J Exp Biol. 199:39–48. 9317295 10.1242/jeb.199.1.39

[bib24] Hahn TP, Cornelius JM, Sewall KB, Kelsey TR, Hau M, Perfito N. 2008. Environmental regulation of annual schedules in opportunistically-breeding songbirds: adaptive specializations or variations on a theme of white-crowned sparrow?. Gen Comp Endocrinol. 157:217–26. 18602554 10.1016/j.ygcen.2008.05.007

[bib25] Hart JS . 1962. Seasonal acclimatization in four species of small wild birds. Physiol Zool. 35:224–36.

[bib26] Hau M . 2001. Timing of breeding in variable environments: tropical birds as model systems. Horm Behav. 40:281–90. 11534993 10.1006/hbeh.2001.1673

[bib27] Hawley DM, Durant SE, Wilson AF, Adelman JS, Hopkins WA. 2012. Additive metabolic costs of thermoregulation and pathogen infection. Funct Ecol. 26:701–10.

[bib28] Hiebert SM . 1991. Seasonal differences in the response of rufous hummingbirds to food restriction: body mass and the use of torpor. The Condor. 93:526–37.

[bib29] Hohtola E . 2012. Thermoregulatory adaptations to starvation in birds. In: Comparative physiology of fasting, starvation, and food limitation. New York (NY): Springer. p. 155–70.

[bib30] Karasov WH, Diamond JM. 1983. Adaptive regulation of sugar and amino acid transport by vertebrate intestine. Am J Physiology. 245:G443–62. 10.1152/ajpgi.1983.245.4.G443 6353941

[bib32] Karasov WH, Pinshow B, Starck JM, Afik D. 2004. Anatomical and histological changes in the alimentary tract of migrating blackcaps (*Sylvia atricapilla*): a comparison among fed, fasted, food-restricted, and refed birds. Physiol Biochem Zool. 77:149–60. 15057725 10.1086/381465

[bib31] Karasov WH, Pinshow B. 2000. Test for physiological limitation to nutrient assimilation in a long-distance passerine migrant at a springtime stopover site. Physiol Biochem Zool. 73:335–43. 10893173 10.1086/316746

[bib33] Kendeigh SC . 1969. Energy responses of birds to their thermal environments. Wilson Bull. 81:441–9.

[bib34] Ketterson ED, King JR. 1977. Metabolic and behavioral responses to fasting in the white-crowned sparrow (*Zonotrichia leucophrys gambelii*). Physiol Zool. 50:115–29.

[bib35] Ketterson ED, Nolan V Jr. 1982. The role of migration and winter mortality in the life history of a temperate-zone migrant, the dark-eyed Junco, as determined from demographic analyses of winter populations. The Auk. 99:243–59.

[bib77_642_263124] Kim, H. Y. (2013). Statistical notes for clinical researchers: assessing normal distribution (2) using skewness and kurtosis. Restor dent endod. 38:52. 23495371 10.5395/rde.2013.38.1.52 PMC3591587

[bib36] King JR, Farner DS. 1966. The adaptive role of winter fattening in the white-crowned sparrow with comments on its regulation. Am Nat. 100:403–18.

[bib37] Klaassen M, Biebach H. 1994. Energetics of fattening and starvation in the long-distance migratory garden warbler, *Sylvia borin*, during the migratory phase. J Comp Physiol B. 164:362–71.

[bib38] Kleiber M . 1947. Body size and metabolic rate. Physiol Rev. 27:511–41. 20267758 10.1152/physrev.1947.27.4.511

[bib39] Lee KA, Karasov WH, Caviedes‐Vidal E. 2002. Digestive response to restricted feeding in migratory yellow-rumped warblers. Physiol Biochem Zool. 75:314–23. 12177834 10.1086/342003

[bib40] Li L, Li B, Li M, Speakman JR. 2019. Switching on the furnace: regulation of heat production in brown adipose tissue. Mol Aspects Med. 68:60–73. 31325458 10.1016/j.mam.2019.07.005

[bib41] Liknes ET, Swanson DL. 2011. Phenotypic flexibility of body composition associated with seasonal acclimatization in passerine birds. J Therm Biol. 36:363–70.

[bib42] Lustick S, Battersby B, Mayer L. 1982. Energy exchange in the winter acclimatized American goldfinch, *Carduelis ( Spinus ) tristis*. Comp Biochem Physiol A Physiol. 72:715–9. 10.1016/0300-9629(82)90240-7 6125311

[bib43] Lv J, Xie Z, Sun Y, Sun C, Liu L, Yu T, Xu X, Shao S, Wang C. 2014. Seasonal plasticity of duodenal morphology and histology in *Passer montanus*. Zoomorphology. 133:435–43.

[bib44] Mcewen BS, Wingfield JC. 2003. The concept of allostasis in biology and biomedicine. Horm Behav. 43:2–15. 12614627 10.1016/s0018-506x(02)00024-7

[bib45] Mercer JG, Moar KM, Logie TJ, Findlay PA, Adam CL, Morgan PJ. 2001. Seasonally inappropriate body weight induced by food restriction: effect on hypothalamic gene expression in male Siberian hamsters. Endocrinology. 142:4173–81. 11564670 10.1210/endo.142.10.8454

[bib46] Michael Romero L . 2002. Seasonal changes in plasma glucocorticoid concentrations in free-living vertebrates. Gen Comp Endocrinol. 128:1–24. 12270784 10.1016/s0016-6480(02)00064-3

[bib47] Morris D . 1956. The feather postures of birds and the problem of the origin of social signals. Behaviour. 9:75–111.

[bib48] Mueller P, Diamond J. 2001. Metabolic rate and environmental productivity: well-provisioned animals evolved to run and idle fast. Proc Natl Acad Sci USA. 98:12550–4. 11606744 10.1073/pnas.221456698 PMC60091

[bib49] Nagy KA . 1987. Field metabolic rate and food requirement scaling in mammals and birds. Ecol Monogr. 57:112–28.

[bib50] Nagy KA . 2005. Field metabolic rate and body size. J Exp Biol. 208:1621–5. 15855393 10.1242/jeb.01553

[bib51] Nowack J, Giroud S, Arnold W, Ruf T. 2017. Muscle non-shivering thermogenesis and its role in the evolution of endothermy. Front Physiol. 8:889. 29170642 10.3389/fphys.2017.00889 PMC5684175

[bib52] Obst BS, Diamond J. 1992. Ontogenesis of intestinal nutrient transport in domestic chickens (*Gallus gallus*) and its relation to growth. The Auk. 109:451–64.

[bib53] Olsen RE, Cox Jr RR, Afton AD, Ankney CD. 2011. Diet and gut morphology of male mallards during winter in North Dakota. Waterbirds. 34:59–69.

[bib54] Pendergast B, Boag D. 1973. Seasonal changes in the internal anatomy of spruce grouse in Alberta. The Auk. 90:307–17.

[bib55] Petit M, Lewden A, Vézina F. 2014. How does flexibility in body composition relate to seasonal changes in metabolic performance in a small passerine wintering at northern latitude?. Physiol Biochem Zool. 87:539–49. 24940918 10.1086/676669

[bib56] Pierce BJ, Mcwilliams SR. 2004. Diet quality and food limitation affect the dynamics of body composition and digestive organs in a migratory songbird (*Zonotrichia albicollis*). Physiol Biochem Zool. 77:471–83. 15286920 10.1086/383503

[bib57] Pyle P . 2022. Identification guide to North American birds, part I. 2nd ed. Point Reyes Station (CA): Slate Creek Press.

[bib58] Robart AR, Watts HE. 2023. Integration of social and temperature cues alters facultative migratory response to declining food availability. Anim Behav. 198:153–64.

[bib59] Rogers CM . 1987. Predation risk and fasting capacity—do wintering birds maintain optimal body-mass. Ecology. 68:1051–61.

[bib60] Stier A, Massemin S, Criscuolo F. 2014. Chronic mitochondrial uncoupling treatment prevents acute cold-induced oxidative stress in birds. J Comp Physiol B. 184:1021–9. 25183199 10.1007/s00360-014-0856-6

[bib62] Swanson DL, Liknes ET. 2006. A comparative analysis of thermogenic capacity and cold tolerance in small birds. J Exp Biol. 209:466–74. 16424096 10.1242/jeb.02024

[bib63] Swanson DL, Vézina F. 2015. Environmental, ecological and mechanistic drivers of avian seasonal metabolic flexibility in response to cold winters. J Ornithol. 156:377–88.

[bib61] Swanson DL . 2010. Seasonal metabolic variation in birds: functional and mechanistic correlates. In: Current ornithology. Vol. 17. New York (NY): Springer. pp. 75–129.

[bib64] Swatson HK, Gous R, Iji PA, Zarrinkalam R. 2002. Effect of dietary protein level, amino acid balance and feeding level on growth, gastrointestinal tract, and mucosal structure of the small intestine in broiler chickens. Anim Res. 51:501–15.

[bib65] Valone TJ, Templeton JJ. 2002. Public information for the assessment of quality: a widespread social phenomenon. Philos Trans R Soc Lond B Biol Sci. 357:1549–57. 12495512 10.1098/rstb.2002.1064 PMC1693063

[bib66] Vernasco BJ, Cornelius JM, Watts HE. 2024. Food and social cues modulate reproductive development but not migratory behavior in a nomadic songbird, the Pine Siskin. J Ornithol. 141:ukae006.

[bib67] Williams JB, Tieleman BI. 2000. Flexibility in basal metabolic rate and evaporative water loss among hoopoe larks exposed to different environmental temperatures. J Exp Biol. 203:3153–9. 11003826 10.1242/jeb.203.20.3153

[bib68] Wingfield JC, Smith JP, Farner DS. 1980. Changes in plasma levels of luteinizing hormone, steroid, and thyroid hormones during the postfledging development of white-crowned sparrows (*Zonotrichia leucophrys*). Gen Comp Endocrinol. 41:372–7. 7409445 10.1016/0016-6480(80)90081-7

[bib69] Witter MS, Cuthill IC. 1993. The ecological costs of avian fat storage. Philos Trans R Soc Lond B Biol Sci. 340:73–92. 8099746 10.1098/rstb.1993.0050

[bib70] Witter MS, Swaddle JP, Cuthill IC. 1995. Periodic food availability and strategic regulation of body mass in the European starling, *Sturnus vulgaris*. Funct Ecol. 9:568–74.

[bib71] Word KR, Austin SH, Wingfield JC. 2022. Allostasis revisited: a perception, variation, and risk framework. Front Ecol Evol. 10:954708.

[bib72] Wu M, Xiao Y, Yang F, Zhou L, Zheng W, Liu J. 2014. Seasonal variation in body mass and energy budget in Chinese bulbuls (*Pycnonotus sinensis*). Avian Res. 5:1–10. 10.11813/j.issn.0254-5853.2014.1.033 PMC5042950 24470452

[bib73] Wurtz MC, Cussen V, Cornelius JM. 2021. The effects of food limitation on behavior, corticosterone, and the use of social information in the red crossbill (*Loxia curvirostr*a). Anim Cogn. 24:1–13. 34047864 10.1007/s10071-021-01520-5

[bib74] Zhang Y, Yang K, Yang P, Su Y, Zheng W, Liu J. 2018. Food restriction decreases BMR, body and organ mass, and cellular energetics, in the Chinese Bulbul (*Pycnonotus sinensis*). Avian Res. 9:1–11.

[bib75] Zheng W-H, Li M, Liu J-S, Shao S-L. 2008. Seasonal acclimatization of metabolism in Eurasian tree sparrows (*Passer montanus*). Comp Biochem Physiol A Mol Integr Physiol. 151:519–25. 18804172 10.1016/j.cbpa.2008.07.009

[bib76] Zheng W-H, Liu J-S, Swanson DL. 2014. Seasonal phenotypic flexibility of body mass, organ masses, and tissue oxidative capacity and their relationship to resting metabolic rate in Chinese bulbuls. Physiol Biochem Zool. 87:432–44. 24769707 10.1086/675439

